# Spotted fever rickettsiae in wild-living rodents from south-western Poland

**DOI:** 10.1186/s13071-017-2356-5

**Published:** 2017-09-06

**Authors:** Ewa Gajda, Joanna Hildebrand, Hein Sprong, Katarzyna Buńkowska-Gawlik, Agnieszka Perec-Matysiak, Elena Claudia Coipan

**Affiliations:** 1 Department of Parasitology, Institute of Genetics and Microbiology, Faculty of Biological Sciences, University of Wrocław, Przybyszewskiego 63/77 Str, 51-148 Wrocław, Poland; 2 Centre for Infectious Disease Control, National Institute for Public Health and Environment, Antonie van Leeuwenhoeklaan 9, 3721 MA Bilthoven, The Netherlands

**Keywords:** *Rickettsia* spp., Rodents, PCR, Poland

## Abstract

**Background:**

Rickettsiae are obligate intracellular alpha-proteobacteria. They are transmitted via arthropod vectors, which transmit the bacteria between animals and occasionally to humans. So far, much research has been conducted to indicate reservoir hosts for these microorganisms, but our knowledge is still non-exhaustive. Therefore, this survey was undertaken to investigate the presence of *Rickettsia* spp. in wild-living small rodents from south-western Poland.

**Results:**

In total, 337 samples (193 from spleen and 144 from blood) obtained from 193 wild-living rodents: *Apodemus agrarius*, *Apodemus flavicollis*, and *Myodes glareolus* were tested by qPCR for *Rickettsia* spp. based on a fragment of *gltA* gene. The prevalence of infection was 17.6% (34/193). Subsequently, the positive samples were analysed by conventional PCR targeting the *gltA* gene fragment. The genus *Rickettsia* was confirmed by sequence analysis in four samples from the blood. In two blood samples from *A. agrarius*, the identified pathogen was *Rickettsia helvetica*. The *Rickettsia* obtained from *A. flavicollis* was assigned to *Rickettsia felis*-like organisms group. One isolate from *A. agrarius* could be determined only to the genus level.

**Conclusions:**

This study shows the presence of *Rickettsia* DNA in tissues of wild-living rodents, suggesting some potential role of these animals in temporarily maintaining and spreading the bacteria in enzootic cycles.

## Background

Species of *Rickettsia* bacteria are classified in the order Rickettsiales (α-Proteobacteria) which contains microorganisms infecting eukaryotic cells. The genus *Rickettsia* is divided into four groups, based on whole-genome analysis data. *Rickettsia bellii* and *Rickettsia canadensis* belong to the ancestral group (AG), for which no pathogenicity was recorded. The typhus group (TG) comprises *Rickettsia typhi* and *Rickettsia prowazekii*. The spotted fever group (SFG) has several bacterial species, including *Rickettsia rickettsii*, *Rickettsia parkeri*, *Rickettsia conorii* and *R*. *helvetica*. The fourth, transitional group (TRG) consists of *Rickettsia akari*, *Rickettsia australis* and *R*. *felis*. Most of the SFG are transmitted by hard ticks of the family Ixodidae and are considered zoonotic. The TG includes rickettsiae transmitted by fleas, lice and mites [1–3]. The tick-borne rickettsioses in Europe are caused by *R*. *conorii* and *R*. *massiliae*, causing Mediterranean spotted fever (MSF), but also by *R*. *slovaca*, *R*. *raoultii* and *R*. *rioja*, causing tick-borne lymphadenopathy (TIBOLA, or *Dermacentor*-borne necrosis erythema and lymphadenopathy, DEBONEL) [4]. Although human infections with two SGF rickettsiae, *R*. *helvetica* and *R*. *monacensis*, were reported in Europe, their pathogenic potential remains controversial [5].

Despite various studies on the occurrence of *Rickettsia* spp. in wild-living small mammals, the role of these animals in maintaining these bacteria is unclear [6–9]. Therefore, we performed a survey on the prevalence of infection with rickettsiae in three rodent species *Apodemus agrarius* (striped field mouse), *Apodemus flavicollis* (yellow-necked mouse) and *Myodes glareolus* (bank vole), trapped in southwestern Poland. We additionally aimed to test detectability of *Rickettsia* DNA in spleen in comparison to blood samples. To the best of our knowledge, this is the first report of the presence of *Rickettsia* spp. in wild-living rodents from Poland.

## Methods

### Sample collection

In 2014, wild-living rodents belonging to the species *A. agrarius*, *A. flavicollis* and *M. glareolus* were live captured. The study areas from which the animals were trapped are located in south-western Poland (Lower Silesia). These are irrigation fields Osobowice, water distribution area Mokry Dwór, the nature reserve “Milicz Ponds” and the Ślęża Landscape Park. Wild-living rodents were live trapped during summer and autumn using Sherman traps and wooden traps, with a piece of fried bread with peanut butter and piece of apple as bait. Approximately 100 traps were used at each site. These were set out at intervals in lines; single traps were placed within 3 m of one another. The trapping period comprised 2–3 days at each site, and traps were inspected in the morning and just before dusk. The captured animals were brought to the laboratory of the Department of Parasitology University of Wroclaw, then were anaesthetized (Isofluranum) and sacrificed by the cervical dislocation. Whole blood, where possible, was taken from the *sinus orbitalis*, collected into 0.001 M EDTA and stored at 4 °C before DNA isolation. Spleen tissues were collected from the same individual and stored frozen before isolation. The animal procedures were approved by the Local Ethics Committee (No. 48/2012).

### Molecular analysis

The DNA was extracted using the following kits for DNA isolation: GeneMATRIX Quick Blood DNA Purification Kit (EURx, Gdansk, Poland) and GeneMATRIX Bio-Trace DNA Purification Kit (EURx) or BIOTRACE Genomic Extraction GPB Mini Kit (GPB GenoPlast Biochemicals, Rokocin, Poland) from blood and spleen samples, respectively. All extraction steps were done in accordance with manufacturer’s instructions. The DNA was stored at -20 °C until analysis for the presence of pathogens. For initial screening of the samples, a duplex qPCR (quantitative real-time polymerase chain reaction) was used. This qPCR assay targeted *Rickettsia helvetica*, SFG, and TG *Rickettsia* using citrate synthase (*gltA*) gene fragments [10, 11]. For *R*. *helvetica* specific qPCR we used the forward primer 5′-ATG ATC CGT TTA GGT TAA TAG GCT TCG GTC-3′, the reverse primer 5′-TTG TAA GAG CGG ATT GTT TTC TAG CTG TC-3′ and the probe 5′-Atto425-CGA TCC ACG TGC CGC AGT-BHQ1-3′. In the second qPCR we used the forward primer 5′-TCG CAA ATG TTC ACG GTA CTT T-3′, the reverse primer 5′-TCG TGC ATT TCT TTC CAT TGT G-3′ and the probe 5′-Atto520-TGC AAT AGC AAG AAC CGT AGG CTG GAT G-BHQ1-3′. The qPCR was performed on a LightCycler 480 Real-Time PCR System (Hoffmann-La Roche, Basel, Switzerland) under the following cycling conditions: 95 °C for 5 min, 60 cycles of 94 °C for 5 s, 60 °C for 35 s, and cooling at 37 °C for 20 s. Samples were considered positive if the C_q_ values were below 45 cycles. To confirm the results, we used conventional PCR also targeting the *gltA* gene fragment of *Rickettsia* spp. The 849 bp product was amplified by using the primers CS409F 5′-CCT ATG GCT ATT ATG CTT GC-3′ and Rp1258n 5′-ATT GCA AAA AGT ACA GTG AAC A-3′ [12, 13]. PCR conditions consisted of an initial denaturation at 95 °C for 15 min, 40 cycles of denaturation at 94 °C for 30 s, annealing at 54 °C for 30 s, extension at 72 °C for 55 s and a final extension at 72 °C for 7 min. Additionally, for specifying the identified *Rickettsia* species, the fragment of outer-membrane protein *OmpB* gene was used. The conventional PCR was carried out with the specific primers: 120–2788 5′-AAA CAA TAA TCA AGG TAC TGT-3′ and 120–3599 5′-TAC TTC CGG TTA CAG CAA AGT-3′ under the following conditions: 94 °C for 3 min, 40 cycles of 95 °C for 30 s, 50 °C for 30 s, 68 °C for 90s and final extension at 68 °C for 7 min [6, 14]. Each qPCR and PCR run included positive and negative controls. Electrophoresis was done in a 2% agarose gel stained with SYBR Gold (Invitrogen, Thermo Fisher Scientific Waltham, MA USA). The positive PCR products were purified with ExoSAP-IT (Thermo Fisher Scientific), and the amplicons were sent for sequencing to Baseclear (Leiden, The Netherlands).

Sequences were compared with corresponding sequences registered in the GenBank database using the NCBI BLAST program (http://blast.ncbi.nlm.nih.gov). Multiple sequence alignment for own sequences of the partial *gltA* gene and similar sequences of *Rickettsia* available in GenBank was conducted using CLUSTAL W implemented in BioEdit program version 7.0.1 [15]. Phylogenetic analysis was performed using MEGA 6.0 software [16].

### GenBank accession numbers

The novel sequences of *Rickettsia* from *A. agrarius* and *A. falvicollis* were deposited in the GenBank database under the accession numbers KY488349 and KY488187, respectively.

## Results

We tested 193 wild-living rodents consisting of 136 striped field mice (*A. agrarius*), 31 yellow-necked mice (*A. flavicollis*), and 26 bank voles (*M*. *glareolus*) captured in 2014. The spleen was collected from all rodents and blood samples from 144 of them (Table 1). *Rickettsia* spp. were detected in 10.4% of the samples (35/337): two samples derived from spleen (1.0%) and 33 samples from blood (22.9%). From one *A. agrarius* captured in the Ślęża Landscape Park, both blood and spleen were positive. The total prevalence of infection was 17.6% (34/193).

**Table 1:** *Rickettsia* spp. detected in rodents (*n* = 193) from Lower Silesia (Poland)

Rodent species	No. positive/no. examined qPCR for *Rickettsia* spp.		No. positive conventional PCR (and sequencing) for *glt*A of *Rickettsi*a spp.	
	Blood	Spleen	Blood	Spleen
*Apodemus agrarius*	25/96	1/136	4 (3)	0
*Apodemus flavicollis*	5/25	0/31	1	0
*Myodes glareolus*	3/23	1/26	0	0
Total	33/144	2/193	5 (4)	0

Following conventional PCR and sequencing on all the qPCR-positive samples, we obtained four sequences that showed similarity with *gltA* of *Rickettsia* spp. (Table 1). Two of them (739 bp) were identical and showed 100% homology with *R*. *helvetica* strain C9P9 (GenBank accession no. U59723) as well as with sequences of *R*. *helvetica* isolated from *Ixodes persulcatus* from Russia (KU310588, KT825960, KM288465) and *Ixodes ricinus* from Hungary (LC060720). These two *R*. *helvetica* isolates originated from blood samples of *A. agrarius* from the Ślęża Landscape Park. Analysis of shorter part of these sequences (341 bp) indicated 100% homology with *R*. *helvetica* obtained in Poland from *I. ricinus* ticks (EU779822, DQ105664) and from *I*. *ricinus* derived from mammals, i.e. *A. flavicollis* (KJ740389) and *Rhinolophus hipposideros* (KJ577821).

**Fig. 1:**
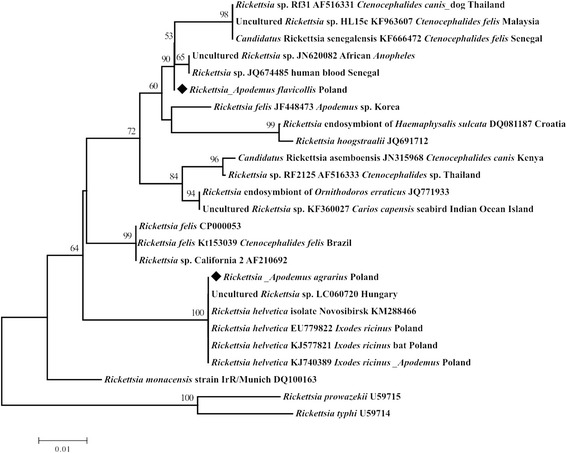
Phylogenetic analysis of *Rickettsia* species identified in this study based on the *gltA* gene. The phylogenetic tree was inferred using the Neighbour-Joining method. The percentage of replicate trees in which the associated taxa clustered together in the bootstrap test (1000 replicates) are shown above the branches. The evolutionary distances were computed using the Maximum Composite Likelihood method

A third sequence, derived from *A. flavicollis* from the Ślęża Landscape Park clustered within the *R. felis*-like organisms (RFLOs) group [9]. This sequence showed 99.3% with *Rickettsia* sp. obtained from *Anopheles gambiae* from Cote d’Ivoire (JN620082), 99.0% with *Rickettsia* sp. Rf31 isolated from *Ctenocephalides canis* derived from a dog in Thailand (AF516331), and Malaysian uncultured *Rickettsia* sp. (KF963607) obtained from *C. felis*, 98.9% similarity with the recently described “*Candidatus* Rickettsia senegalensis” (KF666472) [17], and only 97.4% with *R. felis* (CP000053, AF210692). Additionally, the sequence of *R. felis* (339 bp) (JF448473) obtained from flea parasitizing on *A. agrarius* in Korea demonstrated 98.8% similarity. Our isolate, as one of the *Rickettsia* strains collected from *Ctenophthalmus solutus* flea from *A. agrarius* in Slovakia [18], showed 96% similarity with partial *gltA* gene of *R. felis* (CP000053) and 98% identity with uncultured *Rickettsia* detected in Africa from *Anopheles* mosquito (JN620082) and from human blood sample (JQ674485). The phylogenetic tree shows the positions of *Rickettsia* obtained from *Apodemus* mice in Lower Silesia (Poland) among recognized *Rickettsia* species and isolates (Fig. 1). Attempts to further specify the identified *Rickettsia* species by PCR amplification of fragments of the *OmpB* gene were not successful.

The fourth sequence, obtained from *A. agrarius* from the nature reserve “Milicz Ponds” remained determined at the genus level and it was not included in the further phylogenetic analysis. BLAST search showed that it shares 73% identity with *R. felis* (KT153040, CP000053) and with *R. hoogstraalii* (KT791209, JQ691712) at 80% cover query.

## Discussion

*Rickettsia* species have been detected in ticks in almost every European country [4, 19–21]. Studies conducted in Poland also showed the presence of *Rickettsia* spp. in ticks. In *I*. *ricinus* infections with *R. helvetica*, *R*. *slovaca*, *R*. *raoultii* and *R*. *monacensis* were reported [22–25], whereas in *Dermacentor reticulatus* ticks, *R*. *raoultii* was found [26, 27]. In Lower Silesia, the area of this study, the occurrence of *I. ricinus*, *I. hexagonus*, *I. trianguliceps* and *D. reticulatus* was confirmed [28–30]. Recently, DNA of *R. helvetica*, *R*. *monacensis* and *R*. *raoultii* were identified from ticks parasitizing dogs and cats in the Wrocław Agglomeration (Lower Silesia) [30].

The reservoir role of wild-living animals, including rodents, in rickettsiae life-cycle is still not clear. Ticks can transovarially and transstadially transmit rickettsiae, and serve as vectors and reservoirs of these pathogens. This phenomenon could suggest that no other hosts are needed to complete the *Rickettsia* life-cycle. However, according to other research, vertebrates are also suspected to be a reservoir of rickettsiae. They may also be accidental hosts or amplifier hosts, as in *R*. *rickettsia* [19, 20]. Several serological and molecular studies have suggested that rodents are susceptible to infection with *Rickettsia* species*.* Experimental infections with *R. slovaca*, *R. sibirica*, *R*. *conorii*, *R*. *acari*, *R. prowazekii*, *R. typhi* and *R. rickettsia* in free-living mice and voles, as well as in laboratory rodents have been carried out in Slovakia and USA [31–33]. The differences observed in antibody response to the various infections were dependent on dosage, route of inoculation, bacteria and the species of the rodent [31, 32]. The aim of another experimental study was comparing the sensitivity of conventional PCR and real-time PCR assays for the detection of rickettsial DNA in tissue samples from *Rickettsia*-infected laboratory rodents. The rickettsial DNA was detected in 37.9% of qPCR samples, but with differences between blood and skin biopsy, i.e. 23.6% and 65.6%, respectively [33].

Research addressing the presence of *Rickettsia* DNA in European rodents in nature conducted in The Netherlands [34], Germany [6], Austria [35], and Slovakia [36] showed a prevalence of *Rickettsia* infection varying from 2.7 to 29%, with *R. helvetica* as the most frequent species. However, in other studies in which tissue samples of rodents and other small mammals were examined for the presence of *Rickettsia* DNA, results were negative [7, 23, 25, 37, 38]. Simultaneously, the presence of rickettsial DNA was revealed in ticks, fleas and mites removed from rodents [18, 23, 25, 34, 36, 37]. The last published research from Slovakia showed that 0.5% of rodents and 5.2% of engorged *I. ricinus* ticks removed from voles and mice were *R. helvetica* and *R. monacensis*-positive, but rickettsial DNA was detected only in *A. flavicollis* specimens, which were not infested by ticks [39]. Although in the recently conducted xenodiagnostic experiment, infected rodents, as *Apodemus* spp. or *Myodes glareolus* were not able to transmit *R. helvetica* or *R. monacensis* to *Ixodes* ticks [8].

Our work provides evidence of the occurrence of rickettsiae in wild-living rodents from Poland. The results are consentient with results of other studies and confirm that infection with *Rickettsia* in rodents in the natural environment can occur. However, it should be noted that an accurate comparison of the prevalence of *Rickettsia* between various studies is difficult, due to the diversity of PCR assays, markers, and tissue utilized to detect the bacteria.

After conventional PCR and sequencing, we confirmed positivity of four samples derived only from blood (Table 1). This can be explained by the high sensitivity of the qPCR and simultaneously the low bacteraemia, which is hard to detect using conventional PCR [24, 36, 40]. The short-lasting bacteremia with *Rickettsia* and the use of insufficiently sensitive methods may be a reason why in some research, DNA of bacteria was not detected in samples from animals parasitized by positive ticks or fleas. The differences in sensitivity of DNA detection using different PCR assays has also been observed in experimental *Rickettsia* infection [33]. The results seem to confirm the short-lasting bacteremia with *Rickettsia* occurring in rodents. The level of detection of *Rickettsia* DNA in experimentally infected laboratory animals was 23.6% in blood and 65.6% in skin samples using qPCR, compared to 1.8% and 12.5% using a conventional PCR technique [33].

Although *I. ricinus* is the main vector of *R. helvetica* [4], this bacterium was identified in fleas removed from mice and voles in the Netherlands [34] and in Slovakia [18]. Moreover, the presence of *R. helvetica* and *R. monacensis* in Laelapidae and Trombiculidae mites collected from small mammals was confirmed by Miťková et al. [36]. *Rickettsia felis* is mainly transmitted by cat flea, but other vectors have been reported in different parts of the world; in Europe, the reservoir of this bacteria is unknown [21]. Recently, intra- and interspecific transmission of *R. felis* between co-feeding arthropods (cat fleas transmitted the bacterium to naïve cat fleas and Oriental rat fleas via flea bite) on a vertebrate host was revealed [41]. Thus, the spreading of rickettsiae among different vectors and the demonstration of co-feeding transmission through a vertebrate host represents a novel transmission paradigm for *Rickettsia* spp. [9]. The reporting of rickettsial DNA in various ectoparasites is not synonymous with the ectoparasite being a competent vector, but the mentioned horizontal transmission could have an implication for the epidemiology of pathogens. Future studies, environmental and experimental, to determine the specific roles of both the arthropod and vertebrate host in acquisition and transmission of *Rickettsia* spp., should be performed [9].

The confirmation of both tick- and flea-borne *Rickettsia* spp. in rodents indicates the role of small mammals as hosts for multiple vectors. Furthermore, rodents are known to act as hosts or reservoirs for numerous pathogens and parasites [42]. Their implication in the circulation of multiple vector-borne microorganisms is, therefore, highly possible.

## Conclusions

To our knowledge, this is the first report of the occurrence and the preliminary molecular characterization of *Rickettsia* spp. in rodents in Poland. Furthermore, the bacterium belonging to *R. felis* group as well as other flea-borne *Rickettsia* spp. have never been identified in Poland to date. This study provides evidence that wild-living rodents can be infected with *Rickettsia* bacteria. Knowledge of rickettsiae biology, ecology and their definitive or reservoir hosts is still non-exhaustive. One of the reasons for this is the difficulty in detecting *Rickettsia* DNA, especially in blood and tissues of potential vertebrate hosts. Further investigations are needed to confirm the role of rodents in maintaining the bacteria in nature. More sensitive methods for the detection of bacteria, including other molecular markers, as well as environmental and experimental studies on both vectors and putative hosts are required to elucidate the transmission of *Rickettsia* spp.

## Abbreviations

AG: The ancestral group
DEBONEL: *Dermacentor*-borne necrosis erythema and lymphadenopathy
MSF: Mediterranean spotted fever
qPCR: Quantitative real-time polymerase chain reaction
RFLOs: *Rickettsia felis*-like organisms
SFG: The spotted fever group
TG: The typhus group
TIBOLA: Tick-borne lymphadenopathy
TRG: The transitional group
